# Design and Implementation of Hybrid CORDIC Algorithm Based on Phase Rotation Estimation for NCO

**DOI:** 10.1155/2014/897381

**Published:** 2014-07-07

**Authors:** Chaozhu Zhang, Jinan Han, Ke Li

**Affiliations:** College of Information and Communication Engineering, Harbin Engineering University, Harbin 150001, China

## Abstract

The numerical controlled oscillator has wide application in radar, digital receiver, and software radio system. Firstly, this paper introduces the traditional CORDIC algorithm. Then in order to improve computing speed and save resources, this paper proposes a kind of hybrid CORDIC algorithm based on phase rotation estimation applied in numerical controlled oscillator (NCO). Through estimating the direction of part phase rotation, the algorithm reduces part phase rotation and add-subtract unit, so that it decreases delay. Furthermore, the paper simulates and implements the numerical controlled oscillator by Quartus II software and Modelsim software. Finally, simulation results indicate that the improvement over traditional CORDIC algorithm is achieved in terms of ease of computation, resource utilization, and computing speed/delay while maintaining the precision. It is suitable for high speed and precision digital modulation and demodulation.

## 1. Introduction

Numerical controlled oscillator (NCO) is an important part of digital downconversion. It is widely used in radar wireless transceiver system and software radio system [1–3]. The main function of NCO is to produce two path sine and cosine data samples with variable frequency, discrete time, and mutually orthogonal. It has an advantage of high frequency precision and fast response.

The traditional implement method of NCO is lookup table and polynomial expansion method. Data accuracy of lookup table method depends on the size of the lookup table ROM. The size of the memory and the precision of phase accuracy are exponential relationship, which enlarges the resource consumption and reduces the processing speed of the system. In [4], it solves this problem by using store content mapping technology of odd-even symmetry to optimize the storage unit and reduce the storage resources to 12.5%. However, under the request of high precision, it still consumes a lot of resources. Polynomial expansion method is a real-time computing method which needs multiplier resources and has certain restrictions on the complexity and speed of the hardware. It is too hard for the two methods to trade off speed, accuracy, and resource. Coordinate rotation digital compute algorithm (CORDIC) is proposed to solve the problem. CORDIC algorithm uses a basic algorithm to replace the complex algorithm. CORDIC algorithm is easy to hardware implementation. It does not require hardware multiplier and all operations are only shift accumulation, which meets the hardware requirements of modular and regularization algorithm requirements.

Along with proposing high speed broadband receiver, the data accuracy and processing speed have a higher request. Under the background, traditional CORDIC algorithm has some inherent drawbacks, such as limited coverage angle and too much pipeline series which increase resource consumption and limit data processing speed. Aiming at these shortcomings, this paper puts forward an efficient pipeline architecture CORDIC algorithm for NCO design.

## 2. Traditional CORDIC Algorithm

**Figure 1:**
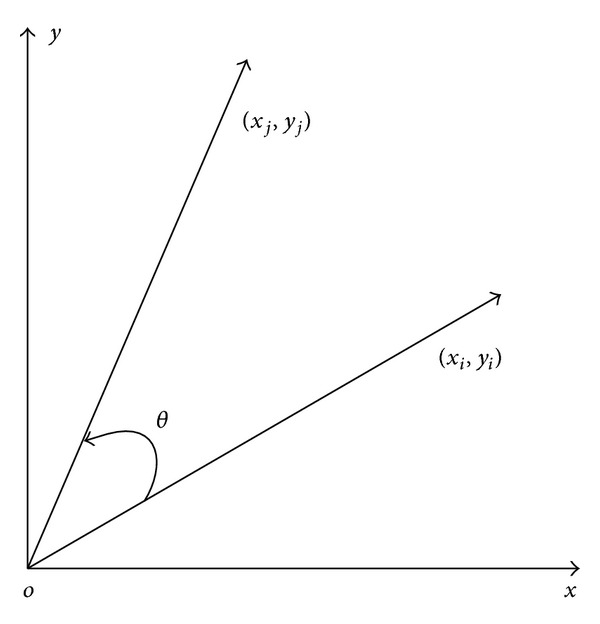
CORDIC vector rotation diagram.

Volder CORDIC algorithm was proposed in 1959, and in 1971, Walther unified the form of the algorithm. Meyer-base realized the algorithm [5, 6], using FPGA implementation for the first time. CORDIC algorithm has been applied in many fields, such as direct digital frequency synthesizer, fast Fourier transform, discrete cosine transform, digital modulation/demodulator, and stream processors [7–10]. According to certain phase, starting point (*x*_*i*_, *y*_*i*_) rotates continuously and approaches the final point gradually. Rotation vector diagram is shown in Figure 1.

In Figure 1, it is easy to get

(1) $$\begin{array}{c} \left[\begin{array}{c} x_{j}\\ y_{j} \end{array}\right]=\left[\begin{array}{cc} \cos\theta & -\sin\theta \\ \sin\theta & \cos\theta \end{array}\right]\left[\begin{array}{c} x_{i}\\ y_{i} \end{array}\right]. \end{array}$$

From the start to the end position, spinning process can be done by several steps and each step only rotates a certain phase:

(2) $$\begin{array}{c} \left[\begin{array}{c} x_{n+1}\\ y_{n+1} \end{array}\right]=\left[\begin{array}{cc} \cos\theta_{n} & -\sin\theta_{n}\\ \sin\theta_{n} & \cos\theta_{n} \end{array}\right]\left[\begin{array}{c} x_{n}\\ y_{n} \end{array}\right]. \end{array}$$

After extracting cos⁡*θ*_*n*_, formula (2) can be expressed as follows:

(3) $$\begin{array}{c} \left[\begin{array}{c} x_{n+1}\\ y_{n+1} \end{array}\right]=\cos\theta_{n}\left[\begin{array}{cc} 1 & -\tan\theta_{n}\\ \tan\theta_{n} & 1 \end{array}\right]\left[\begin{array}{c} x_{n}\\ y_{n} \end{array}\right]. \end{array}$$

In order to simplify the hardware implementation, every operation sets each rotation phase to *θ*_*n*_ = arctan(2^−*n*^). The total rotation phase is *θ* = ∑*S*_*n*_ *θ*_*n*_. So tan*θ*_*n*_ = *S*_*n*_2^−*n*^. Formula (3) can be expressed as follows:

(4) $$\begin{array}{c} \left[\begin{array}{c} x_{n+1}\\ y_{n+1} \end{array}\right]=\cos\theta_{n}\left[\begin{array}{cc} 1 & -S_{n}2^{-n}\\ S_{n}2^{-n} & 1 \end{array}\right]\left[\begin{array}{c} x_{n}\\ y_{n} \end{array}\right]. \end{array}$$

From formula (4), in addition to the cos⁡*θ*_*n*_ coefficient, the operation is simple shift and addition.

In the final result, cos⁡*θ*_*n*_ can be eliminated by multiplying a known constant. For example, *P*, the number of iterations is 16 and |*θ*| ≤ *π*/4. *K* can be expressed as follows:

(5) K=∏n=116cos⁡θn=∏n=116cos⁡(arctan(2−n))=∏n=116(1−2−2i)−1/2.

In the phase rotation process, approximative rotational iterative formula is

(6) $$\begin{array}{c} \left[\begin{array}{c} x_{n+1}\\ y_{n+1} \end{array}\right]=\left[\begin{array}{cc} 1 & -S_{n}2^{-n}\\ S_{n}2^{-n} & 1 \end{array}\right]\left[\begin{array}{c} x_{n}\\ y_{n} \end{array}\right]. \end{array}$$

Parameter *z* is used to judge when the iteration is over: *z*_*n*+1_ = *z*_*n*_ − *θ*_*n*_, *z*_0_ = *θ*. When *z*_*n*_ < *θ*, *S*_*n*_ = −1. When *z*_*n*_ ≥ *θ*, *S*_*n*_ = +1. If the initial value is (*x*_*i*_, *y*_*i*_) = (*x*_0_, *y*_0_) = (*K*, 0), (*x*_*n*_, *y*_*n*_) of *P*th iteration will converge to (cos⁡*θ*, sin*θ*). The phase convergence satisfies the CORDIC convergence theorem [6]. The constant scaling factor *K* is fixed and can be precomputed as long as the precision *N* is determined. After analysis of traditional CORDIC algorithm calculation accuracy, the iteration number and phase precision are expressed as follows:

(7) $$\begin{array}{c} P\geq -\text{lo}{\text{g}}_{2}\left[\tan\left(\Delta \theta_{\min}\right)\right], \end{array}$$

where Δ*θ*_min⁡_ = 2*π*/2^*N*^ and the input phase data width is *N*.

## 3. Hybrid CORDIC Algorithm Based on Phase Rotation Estimation

Common operation structures are iteration, pipeline, and differential CORDIC algorithm. Iterative structure occupies less hardware resources, but the processing data efficiency is low. Although the pipeline structure occupies more hardware resources, it can improve the throughput. Based on the two realization structures, implementation schemes have parallel pipelines, hybrid rotation CORDIC, angle encoding method, and so forth [11, 12]. The work in [13] puts forward the way of prediction rotation direction. The algorithm, applied in error analysis and elimination, has the advantages of fast speed. But it does not optimize hardware structure. Using the structure of the parallel hybrid CORDIC algorithm, the prediction scheme of [14] is more regular and simpler compared to previous approaches, which can reduce the number of iterations by more than 50 percent. However, the judgment of rotation direction is not optimized, which increases latency time and resources, so that it affects the throughput. The work in [15] puts forward a modified hybrid CORDIC algorithm and improved the precision of output data, but the method is more complex. Trading off the disadvantages of the above methods and advantages of pipeline structure and iterative structure, this paper simplifies the CORDIC algorithm further. By using the arctangent function property, it reduces the rotating judgment and add-subtract unit operation.

In this paper, attention is focused mainly on techniques that reduce the number of iterations, while keeping the low latency. The hybrid CORDIC algorithm based on phase rotation estimation is presented in this section, which can be addressed by digit-on-line pipelined CORDIC circuits and repetitive multiple accumulations architecture.

### 3.1. Rotation Phase Estimation

Assuming that the input phase length of CORDIC algorithm is *N* and pipeline series is *P*, rotation phase *θ* can be represented as follows:

(8) $$\begin{array}{c} \theta =\overset{P}{\underset{n=1}{\sum }}S_{n}\theta_{n}=\overset{P}{\underset{n=1}{\sum }}S_{n}\arctan\left(2^{-n}\right), \end{array}$$

where *S*_*n*_ = ±1. It is noted that the initial value of *n* is 1, and the reason is that we restrict the rotation angle within the range |*θ*| ≤ *π*/4 in the application example of NCO.

With the increase of rotational coefficient *n*, arctan(2^−*n*^) gets close to 2^−*n*^. When *n* ≥ 1, 2^−*n*^ > arctan(2^−*n*^). Error is *ε*_*n*_ = 2^−*n*^ − arctan(2^−*n*^). Arctangent function is developed through the tailor equation:

(9) εn=2−n−[2−n−13(2−n)3+15(2−n)5−⋯]=13(2−n)3−15(2−n)5+⋯,

where *ε*_*n*_ < (1/3)2^−3*n*^. The minimum phase value is 2^−*N*^. In the process of phase rotation, when the error estimate is (1/3)2^−3*n*^ ≤ 2^−*N*^, error generated by estimated value 2^−*n*^ can be ignored. The range of *n* is

(10) $$\begin{array}{c} n\geq \frac{N-\text{lo}{\text{g}}_{2}3}{3}. \end{array}$$

When *n* ≥ *m* = ⌈(*N* − log_2_3)/3⌉, arctan(2^−*n*^) ≈ 2^−*n*^. Through (7), pipeline series of CORDIC algorithm is *P* ≥ *N* − 2. The less the pipeline series are, the faster the speed is. When *P* = *N* − 2, we define the hybrid radix set:

(11) $$\begin{array}{c} \theta =\overset{N-2}{\underset{n=1}{\sum }}S_{n}\theta_{n}=\overset{m}{\underset{n=1}{\sum }}S_{n}\arctan\left(2^{-n}\right)+\overset{N-2}{\underset{n=m+1}{\sum }}S_{n}2^{-n}. \end{array}$$

After iterating *m* + 1 times, the sum of residual rotation phase is ∑*θ*_*n*_, as shown in formula (12):

(12) ∑θn=∑n=m+1N−2Sn2−n=Sm+12−m−1+Sm+22−m−2⋯+SN−22−N+2<2−m.

The actual residual phase is *z*_*m*+1_. According to the traditional CORDIC algorithm theory, *z*_*m*+1_ ≈ ∑*θ*_*n*_. So *z*_*m*+1_ < 2^−*m*^. When the (*m* + 1)th rotation begins, the new rotation phase ${\tilde{\theta }}_{m+1}$ is *ϕ*_*m*+1_, where the absolute value of *z*_*m*+1_ is *ϕ*_*m*+1_. Thus $\tan{\tilde{\theta }}_{m+1}={\tilde{S}}_{m+1}{\tilde{\theta }}_{m+1}$. When *z*_*m*+1_ < 0, ${\tilde{S}}_{m+1}=-1$. When *z*_*m*+1_ ≥ 0, ${\tilde{S}}_{m+1}=+1$. Taking it into formula (3),

(13) $$\begin{array}{c} \left[\begin{array}{c} x_{m+2}\\ y_{m+2} \end{array}\right]=\cos\phi_{m+1}\left[\begin{array}{cc} 1 & -{\tilde{S}}_{m+1}{\tilde{\theta }}_{m+1}\\ {\tilde{S}}_{m+1}{\tilde{\theta }}_{m+1} & 1 \end{array}\right]\left[\begin{array}{c} x_{m+1}\\ y_{m+1} \end{array}\right]. \end{array}$$

After the rotation, the residual phase is 0. It shows that *x*_*m*+2_ and *y*_*m*+2_ are the output of cosine data and sine data.

### 3.2. Rotation Function Optimization and Error Analysis

In order to obtain cosine data from the new pipeline process, we put forward unidirectional rotation method to reduce the comparator and choose addition or subtractor. ${\tilde{\theta }}_{m+1}$ should be expressed firstly. *N*-bits input phase needs to iterate *m* times. The results can be expressed as *W*. The residual phase at this time is *z*_*m*+1_. ${\tilde{\theta }}_{m+1}$ is expressed as follows:

(14) $$\begin{array}{c} T=\overset{N-2}{\underset{i=1}{\sum }}A_{i}2^{i}=A_{N-2}2^{N-2}+A_{N-3}2^{N-3}+\cdots +A_{1}2^{1}, \end{array}$$

where *A*_*i*_ = 1 or 0. When *W*[*N* − 2] = 0, *A*_*i*_ = *W*[*i*]. When *W*[*N* − 2] = 1, *A*_*i*_ is that *W*[*i*] flips every bit and adds 1:

(15) $$\begin{array}{c} {\tilde{\theta }}_{m+1}=\frac{2\pi T}{2^{N}}=\frac{\tilde{T}}{2^{N}}. \end{array}$$

From formula (15), $\tilde{T}$ is unknown. In the hardware implementation, $\tilde{T}$ needs to be expressed as follows:

(16) T~=∑i=1NBi2i=2πT=∑i=1NAi2i(22+2+2−2+2−5+2−9),

where *B*_*i*_ = 1 or 0. Taking all figures of $\tilde{T}$ into (15),

(17) θ~m+1=∑i=1N−m−1Bi2i−N=BN−m−12−m−1+⋯+B222−N+B121−N.

Uniting formulas (13) and (17),

(18) $$\begin{array}{c} \left[\begin{array}{c} x_{m+2}\\ y_{m+2} \end{array}\right]=\left[\begin{array}{c} x_{m+1}-{\tilde{S}}_{m+1}\cdot \overset{N-m-1}{\underset{i=1}{\sum }}B_{i}2^{i-N}\cdot y_{m+1}\\ {\tilde{S}}_{m+1}\cdot \overset{N-m-1}{\underset{i=1}{\sum }}B_{i}2^{i-N}\cdot x_{m+1}+y_{m+1} \end{array}\right], \end{array}$$

where ${\tilde{S}}_{m+1}=\pm 1$ and *B*_*i*_ = 1 or 0.

CORDIC algorithm of efficient pipeline uses $\left\{\arctan2^{-1},\ldots ,\arctan2^{-m-1},{\tilde{\theta }}_{m+1}\right\}$ instead of the traditional rotation phase {arctan2^−1^,…, arctan2^−*m*−1^,…, arctan2^−*P*^}. The last set of rotation phase can be expressed as binary. Rotation direction is obtained directly from the last set. One more shift and add operation reduces *N* − *m* − 3 rotation times. Under the premise of ensuring phase and data accuracy, it reduces the resource consumption and improves the operation speed. Finally, with fewer lines series, constant coefficient is as follows:

(19) $$\begin{array}{c} \hat{K}=\overset{m+1}{\underset{n=1}{\prod }}\cos\left(\arctan\left(2^{-n}\right)\right). \end{array}$$

At this time set the initial value (*x*_0_, *y*_0_) to $\left(\hat{K},0\right)$. According to the above process, (*x*_*m*+2_, *y*_*m*+2_) converges to (cos⁡*θ*, sin*θ*).

The cosine error of this algorithm can be divided into three parts:

1. the quantization errors are caused due to the limited word length,
2. limited phase word length leads to approximation error,
3. the phase estimation gives rise to the rotation estimation error.

Quantization error is in an inverse ratio to word length and output word length is set by pipeline series. The more the pipeline series are, the lower the quantization error is. But the increase of pipeline series will lead to resources consumption. So according to the data figure, it is necessary to trade off pipeline series and quantization error. Considering the hardware consumption, computing speed, and precision, [7] proposes the optimization method of data bits and pipeline series. According to [16], the quantization error consists of two parts, the quantization error produced before and this time. It can be expressed as follows:

(20) $$\begin{array}{c} \left|E_{n}\right|\leq \left|\left|e_{n}\right|+\overset{n-1}{\underset{i=0}{\sum }}\left(\overset{n-1}{\underset{j=i}{\prod }}S_{j}\right)\left|e_{i}\right|\right|, \end{array}$$

where **E**_*n*_ is the sum of quantization error and **e**_*n*_ is the *n*th phase rotation quantization error with *S*_*j*_ = ±1.

When the output data is *N* and **e**_*i*_ = [*e*_*xi*_ *e*_*yi*_]^*T*^, |*e*_*xi*_| ≤ *ε*, |*e*_*yi*_| ≤ *ε*, *ε* = 2^−*N*−1^, |**e**_*i*_| can be expressed as follows:

(21) $$\begin{array}{c} \left|e_{i}\right|=\sqrt{e_{xi}^{2}+e_{yi}^{2}}\leq \sqrt{2}\times 2^{-N-1}. \end{array}$$

According to formula (7), when phase length is *N*, phase resolution is *φ* = 2*π*/2^*N*^. Approximation error produced by limited phase word length can be expressed as follows:

(22) $$\begin{array}{c} \left|A\right|=\frac{\left|V-V^{\prime }\right|}{\left|V^{\prime }\right|}\leq 2\sin\left(\frac{\Delta \theta }{2}\right)\leq \Delta \theta \leq \varphi , \end{array}$$

where *V* is the actual value and *V*′ is the error value. Δ*θ* is the difference between real phase and approximate phase. In the final rotating phase estimate, rotating phase arctan(2^−*n*^) is instead of 2^−*n*^. Arc value of 2*π* can be replaced only by binary values similarly. In formula (16), the generated error can be expressed as follows:

(23) |B|=∑i=m+2N−3(2−i−arctan(2−i))+2π−6.2832≈∑i=m+2N−3(2−i−arctan(2−i))+2×10−5.

## 4. The FPGA Design and Implementation of NCO

### 4.1. High Speed and Precision NCO Structure

**Figure 2:**
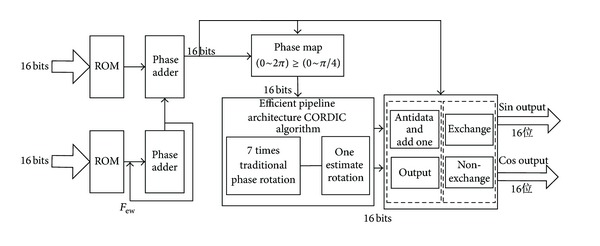
High speed and precision NCO structure.

This paper adopts efficient pipelining structure CORDIC algorithm for high speed and high precision NCO. Its structure is shown in Figure 2. We take 16-bit phase control words as an example. Firstly, input is a 16-bit phase control word and 16-bit frequency control word. Secondly, through the phase accumulator and phase adder, the output is 16-bit phase value. Phase map generates the {0, *π*/4} phase. Thirdly, the shift-add efficient pipelining structure processes phase data. Finally according to the previous mapping relation, 16-bit sine and cosine data can be generated.

The range *θ* of rotation angle value is {−44.855°, 44.855°} and approximates to {−*π*/4, *π*/4}. It does not meet the {0,2*π*} scope of phase. Before 16-bit phase values are sent into the algorithm, cosine function property can judge the highest, second highest, and third highest bit. According to certain mapping relation, the highest 3 bits of 16-bit phase value and phase can be reduced to 3′*b*000 and {0, *π*/4}, respectively. The highest bit controls sine data symbol. If the bit is 1, the algorithm flips the sine data and adds 1. On the other hand, the algorithm does not process input data. The highest bit and second highest bit control cosine data symbol. If they are different, the algorithm flips sine data and adds 1. Otherwise, it remains to be the input data. The second highest bit and third highest bit control the location of cosine data and sine data. If they are different, the algorithm exchanges cosine data and sine data. Or else it remains to be the input data.

### 4.2. Internal Architecture Design and the Major Implementation Steps

**Figure 3:**
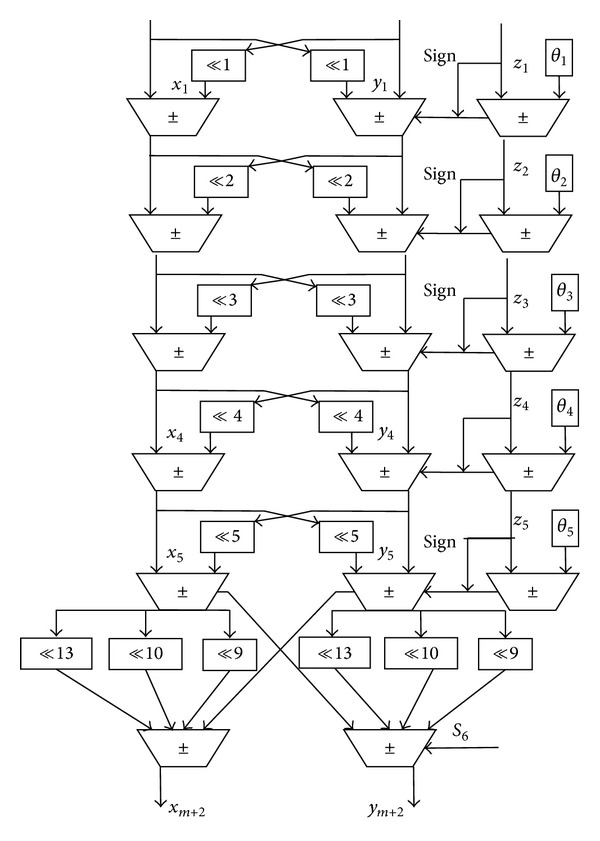
Phase rotation estimation based on pipeline structure.

According to formula (10), our algorithm needs *m* = ⌈(16 − log_2_3)/3⌉ = 5 times for traditional phase rotation and one time for rotation phase estimation. If ${\tilde{\theta }}_{6}=2^{-9}+2^{-10}+2^{-13}$, the pipeline structure is shown in Figure 3. Each level only needs three adder-subtractors, two or six phase shift registers, and a phase coefficient memory and reduces more than a half of the rotation phase judgment and shift operation. For reducing the critical path in the pipelined implementation of traditional CORDIC, the differential CORDIC (D-CORDIC) algorithm based on digit-on-line pipelined CORDIC circuits [17] can be used to achieve higher throughput and lower pipeline latency. D-CORDIC algorithm is equivalent to the usual CORDIC in terms of accuracy as well as convergence. The system architecture uses parallel and pipeline differential CORDIC architecture to reduce latency and improve throughout. Digit-on-line pipelined CORDIC circuits take place of continuous phase accumulation in Figure 3.

From what has been discussed above, the major steps of our algorithm are as follows.

**Step 1 .**

Phase rotation is limited in the range of {−*π*/4, *π*/4}.

**Step 2 .**

Traditional or differential CORDIC algorithm implements partial phase rotation.

**Step 3 .**

Using a relatively simple prediction scheme, we divide original CORDIC rotations into the lower part and the higher part.

**Step 4 .**

Differential CORDIC or traditional architecture is proposed to compute rotation direction. The lower part is computed by continuous accumulation or online architecture [18] based on differential CORDIC and the higher part is predicted by rotation phase estimation.

**Step 5 .**

According to phase mapping relationship, the required high precision and high speed cosine data is produced.

### 4.3. Simulation Results

Table 1 compares the delay of some CORDIC rotation methods. Our proposed algorithm could obtain good performance in delay and resource.

To compare our pipeline CORDIC algorithm with other previously proposed methods fairly, we assume CSA is universal adder in all algorithms and fast carry-propagate adders (CPA) are used in the last stage to take carry-save forms back to the input initial phase value.

In [13], the first *m* iterations use the traditional continuous comparison method, the same as the traditional CORDIC. The delay increases logarithmically with the maximum number of shifts. If the delay of carry-propagate adder (CPA) is ⌈log_2_ *N*⌉ · *T*_*FA*_, the latency of (*N* − *m*) iterations increases linearly with the word length and the delay is (4*N*/3) · *T*_*FA*_.

Based on the calculation method above, the traditional CORDIC based on pipeline architecture has the delay of ⌈log_2_ *N*⌉ · *T*_*FA*_ · *N*.

Unlike the above methods, our proposed method reduces the number of iterations and simplifies the *Z* datapath. The first iterations still adopt the traditional CORDIC algorithm where a delay of ⌈log_2_ *N*⌉ · *T*_*FA*_ is assumed for an *N*-bit CPA. The accumulations of final iteration use repetitive multiple accumulations architecture [19], which has much higher throughput and less delay compared with serial accumulator and pipelined adder based on carry-save addition as well. The last iteration increases linearly and the delay is (4*K*/3) · *T*_*FA*_, where *K* is the full-adder number for the accumulations based on adder-tree architecture.

According to the structure shown in Figure 2, traditional pipeline structure and efficient pipeline structure based on rotation phase estimation are implemented by verilog language, respectively. Hardware platform is a Cyclone II series EP2C8Q208C8 chip and software platform is in Quartus II of Altera company. Modelsim 10.0 simulation software tests the experience result. Firstly, the input frequency control word, phase control word, and clock frequency are set to 16′*h*1999, 16′*d*0, and 100 MHz. Output frequency is 10 MHz. Compared to the use of resources, the result can be expressed in Table 2.

Through the comparison in Table 2, our proposed algorithm reduced resource obviously.

**Figure 4:**
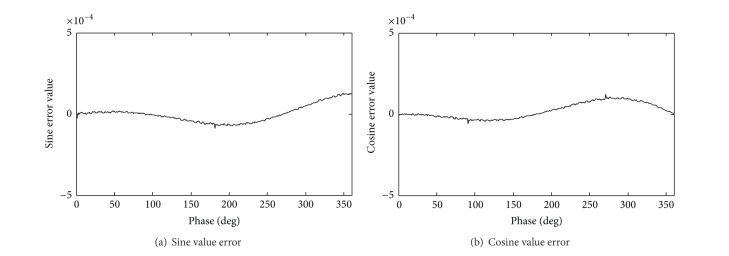
Sine and cosine error statistics of traditional pipeline structure.

**Figure 5:**
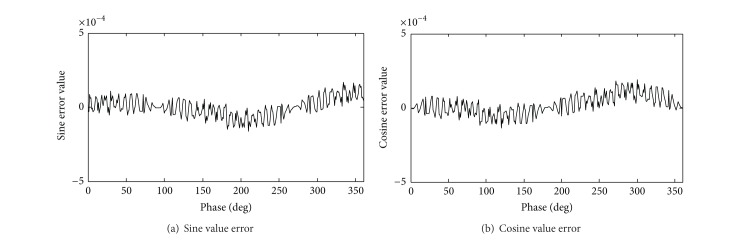
Sine and cosine error statistics of ours.

**Figure 6:**
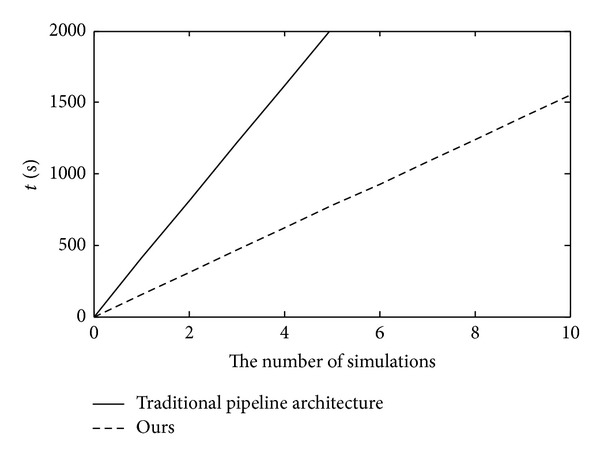
The runtime of algorithms' comparison.

This algorithm precision is the same as traditional CORDIC algorithm, $\Delta {\hat{\theta }}_{\min}=2\pi /2^{N}$. The input frequency control word, phase control word, and clock frequency are set to 16′*h*00*B*6 and 16′*d*0. The output frequency is 0.3125 MHz. Compared with the theoretical value and experiment value, the error statistic is shown in Figures 4 and 5. The simulation runtime of our proposed algorithm costs less than the traditional CORDIC algorithm in Figure 6.

Compared with Figures 4 and 5, our proposed algorithm has the larger error volatility, while the two kinds of the algorithm error will be controlled in (−5 × 10^−4^, 5 × 10^−4^).

**Figure 7:**
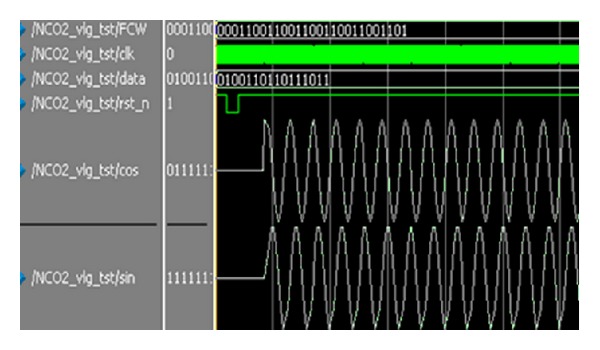
NCO simulation waveform.

Though our algorithm structure reduces logic unit, it guarantees the cosine data accuracy.  Figure 7 shows the NCO simulation waveform of efficient pipeline structure.

It is necessary to obtain efficient bits of phase, optimum iteration number, and data width. We do the above experiment 200 times. The random angle value is restricted from 0 to 45°. When the iteration number is 5~8 and the series of data width are 15, 16, 18, and 21, we can obtain the effective bits. The relationship of effective bit number with iteration times and data width is shown in Table 3. The data unit is degree.

The algorithm error will be controlled in (−5 × 10^−4^, 5 × 10^−4^), when the iteration number is greater than 6. The experimental results show that the effective bit number is 13. Through calculating the minimum number of microrotation, the effective bit number is generally seven greater than iteration number. The calculation of total quantization errors could be calculated through this method.

## 5. Conclusion

In this paper, the hybrid CORDIC algorithm based on phase rotation estimation is proposed to design NCO. In the case of assuring the high precision output, the efficient CORDIC algorithm reduces more than a half of the rotation phase judgment and shift operation. Resource consumption, operation speed, and system delay have much better performance than traditional CORDIC algorithm. In terms of electronic countermeasures, it has a certain practicality. The algorithm has been successfully used in high speed broadband ADS-B receiver and shows good performance.

## Figures and Tables

**Table 1:** Comparison of resource use.

Algorithm	Delay		
	*N* = 16	*N* = 24	*N* = 32
Traditional pipeline structure	64*T*_*FA*_	110*T*_*FA*_	160*T*_*FA*_
Hybrid CORDIC algorithm	43*T*_*FA*_	72*T*_*FA*_	96*T*_*FA*_
Ours	26*T*_*FA*_	37*T*_*FA*_	72*T*_*FA*_

**Table 2:** Algorithm resource use comparison.

Algorithm	Resource		
	Logic unit	Register	Storage size
Traditional pipeline structure	1177	754	63
Hybrid CORDIC algorithm	1034	576	26
Ours	819	393	26

**Table 3:** Relationship of effective bit number with iteration number and data width.

	Data width (iteration number)			
	14 (5)	16 (6)	18 (7)	21 (8)
Estimated value	22.137	22.137	22.137	22.137
Simulated value	23.672	22.458	22.281	22.132
